# The association between dementia and the risk of hypoglycaemia events among patients with diabetes mellitus: a propensity-score matched cohort analysis

**DOI:** 10.3389/fmed.2023.1177636

**Published:** 2023-07-05

**Authors:** Alaa A. Alsharif, Ian C. K. Wong, Tian Ma, Wallis Lau, Meshari Alhamed, Hassan Alwafi, Li Wei

**Affiliations:** ^1^Department of Pharmacy Practice, Faculty of Pharmacy, Princess Nourah Bint Abdulrahman University, Riyadh, Saudi Arabia; ^2^Research Department of Practice and Policy, University College London School of Pharmacy, London, United Kingdom; ^3^Department of Pharmacology and Pharmacy, Li Ka Shing Faculty of Medicine, Centre for Safe Medication Practice and Research, The University of Hong Kong, Hong Kong, China; ^4^Department of Emergency Medicine, Ministry of National Guard Health Affairs, Riyadh, Saudi Arabia; ^5^King Abdullah International Medical Research Center, Riyadh, Saudi Arabia; ^6^Faculty of Medicine, Umm Al Qura University, Mecca, Saudi Arabia

**Keywords:** dementia, diabetes, hypoglycaemia, database, cohort, risk factors

## Abstract

**Background:**

Hypoglycaemia commonly occurs in patients diagnosed with diabetes mellitus (DM) and dementia. The impact of dementia on hypoglycaemic events is controversial. Thus, we evaluated whether dementia increases the risk of hypoglycaemic events in older patients diagnosed with DM.

**Design:**

A retrospective cohort study.

**Setting:**

We used the IQVIA Medical Research Data (IMRD-UK) database (formerly known as the THIN database).

**Participants:**

All patients aged ≥55 years and diagnosed with DM who were prescribed at least two prescriptions of antidiabetic medication between 2000 and 2017. Two groups of patients, dementia and non-dementia group, were propensity-score (PS) matched at 1:2. The risk of hypoglycaemia was assessed through a Cox regression analysis.

**Main outcome and measures:**

Hypoglycaemic events were determined during the follow-up period by Read codes.

**Results:**

From the database, 133,664 diabetic patients were identified, with a mean follow-up of 6.11 years. During the study period, 7,762 diabetic patients diagnosed with dementia were matched with 12,944 diabetic patients who had not been diagnosed with dementia. The PS-matched Cox regression analysis showed that patients diagnosed with dementia were at a 2-fold increased risk for hypoglycaemic events compared with those not diagnosed with dementia (hazard ratio [HR], 2.00; 95% CI, 1.63–2.66). A similar result was shown for a multivariable analysis using all patient data (adjusted HR, 2.25; 95% CI, 2.22–2.32).

**Conclusion:**

Our findings suggest that diabetic patients with a diagnosis of dementia have a statistically significant higher risk of experiencing hypoglycaemia.

## Background

Hypoglycaemia is defined as a condition where blood glucose falls below the normal level, i.e., less than 70 mg/dL (1). It is the most common side effect in diabetes mellitus management and becomes a barrier to effective glycaemic control (2, 3). Several factors may contribute to the development of hypoglycaemia; they can be categorized into three: medication use, comorbid diseases, and individual factors.

Drug-induced hypoglycaemia is frequently experienced by diabetic patients, particularly those who are using insulin analogues and sulfonylureas, compared to other classes of antidiabetic medications (4–6). Other non-diabetes medications associated with hypoglycaemia are quinolones, pentamidine and angiotensin-converting enzyme inhibitors, and angiotensin receptor blockers (7). Comorbid diseases are considered to be the second category that may contribute to the occurrence of hypoglycaemia including dementia, heart failure, malignancies, renal and liver failure, and infections (2, 8). Finally, individual factors that played a role in the occurrence of hypoglycaemia were the patient’s age, being a woman, low body mass index, diabetes mellitus type, history of hypoglycaemia, glycaemic control, and malnutrition (2, 8).

Hypoglycaemic events varied in its severity. It may cause acute and potentially fatal events that cannot be managed easily at home by patients themselves or by family members/carers, and in severe cases, it may require further assistance (9). These hypoglycaemic events may not only impact the patient’s daily activities but also cause serious morbidity. They increase the risk of falls, cognitive impairment, and cardiovascular and cerebrovascular complications which can lead to death (10).

Cognitive impairment, including dementia or milder forms of dysfunction, may delay the recognition of the warning symptoms of hypoglycaemia which is fundamental to effective self-management and the prevention of progression in severity (11, 12). An increased risk of hypoglycaemia has been observed in elderly patients diagnosed with dementia (13).

In this study, we aim to examine the association between dementia and the risk of developing hypoglycaemia among the DM population, and our findings might support and extend the previous literature that dementia is an important risk factor for the development of hypoglycaemia.

## Methods

### Data source and study design

This was a population-based retrospective cohort study that used the IMRD-UK database (formerly known as the THIN database). IMRD-UK is a large electronic patient data recorded by general practitioners (GPs) during routine clinical practice and currently has anonymised clinical data for 13 million patients registered with 744 general practices across the UK (14). The IMRD-UK Data covering approximately 6% of the UK population are largely representative of the UK population in terms of age, sex, and diabetes and dementia diagnosis.

IMRD-UK has been widely used for population-based, epidemiological research and has previously been used in multiple studies for the population diagnosed with DM and/or dementia (15–17).

### Ethical approval

Ethical approval was obtained from the Scientific Review Committee (SRC reference number: 18THIN054).

### Study population

All patients aged 55 years or over who had prevalent DM at baseline or who developed DM during the follow-up period, who have at least two antidiabetic prescriptions and registered within IMRD-UK were identified and followed up from January 2000 to 26 September 2017. Patients were divided into two groups according to the presence of dementia diagnosis. Patients who had dementia at baseline or developed dementia were placed in the dementia group and defined as the exposed group. The non-exposed group were diabetic patients without dementia.

**Figure 1:**
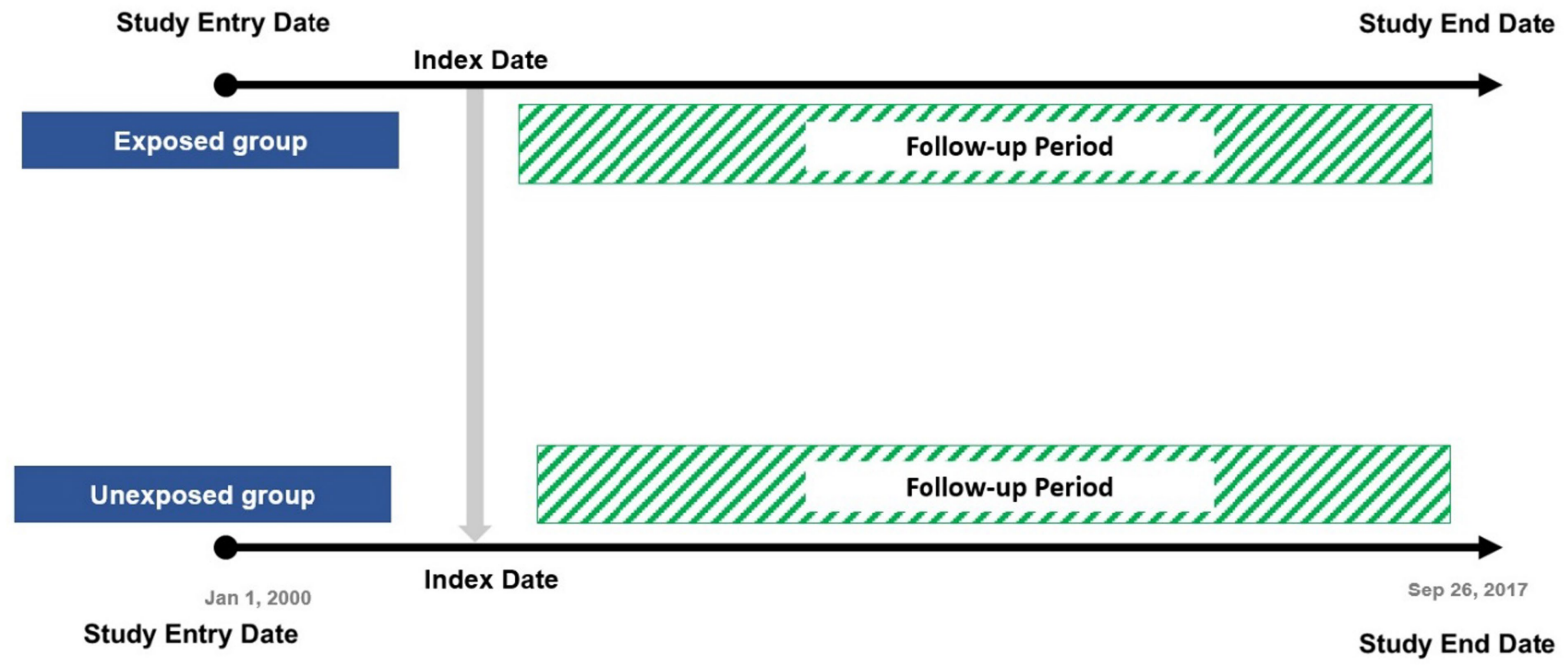
Study design.

The date of the latest first record between DM diagnosis and dementia diagnosis (coexistence of both diseases) during the follow-up was defined as the index date for each exposed participant and was used to assign an index date for the non-exposed group. Patients were censored if they experienced hypoglycaemia (an outcome event after the index date), died, or left their general practice during the study period (Figure 1).

### Measurements

DM diagnoses including type 1 and type 2, dementia, and hypoglycaemia were identified based on the Read Codes.

Any diagnostic code for DM or two records of any prescribed antidiabetic medication were used to identify diabetic patients. Dementia diagnosis was identified by any record of dementia diagnosis or a record of any prescribed anti-dementia medication. We included all records of hypoglycaemic events experienced by patients during the study period. The Read codes used in this cohort were obtained from previously published code lists (18) and published studies (17, 19).

### Covariates

The baseline characteristics covariates included age, sex, body mass index (BMI) (categorized into five categories: BMI < 18.5, BMI = 18.5–24.9, BMI = 25–29.9, BMI = 30–39.9, and BMI ≥ 40), smoking status (categorized into three categories: non-smoker, ex-smoker, and smoker), alcohol consumption (categorized into three categories: non-drinker, ex-drinker, and drinker), HbA1c (categorized into four categories: HbA1c < 5.7, HbA1c = 5.7–6.4, HbA1c = 6.5–13.9, and HbA1c ≥14), and diabetes duration and history of hypoglycaemic events at baseline or prior to the study entry date. Chronic comorbidities included diabetes microvascular complications (including neuropathy, nephropathy, retinopathy, and diabetic foot), hypertension (HTN), myocardial infarction (MI), arrhythmias, heart failure (HF), chronic obstructive pulmonary disease (COPD), chronic kidney disease (CKD), depression, cerebrovascular disease (CVD), lipoedema, and obesity. Co-prescribed medications included angiotensin-converting enzyme inhibitors (ACEI) or angiotensin receptor blockers (ARBs), beta-blockers (BB), calcium channel blockers (CCB), antiarrhythmic medication, statins, aspirin, antidepressants, and anti-dementia medication. Antidiabetic medications were classified according to the British National Formulary (BNF) (20) based on their therapeutic classes: insulin, biguanides (metformin), sulfonylureas (SFU), meglitinides (MEG), thiazolidinedione (TZD), dipeptidyl peptidase-4 inhibitors (DDP-4), sodium-glucose cotransporter-2 inhibitors (SGLT-2), glucagon-like peptide-1 receptor agonists (GLP-1), and acarbose. Chronic comorbidities were measured over the 12-month period prior to the index date (i.e., study entry date). However, medication use was assessed over the 6-month period preceding the index date.

### Outcomes

The study outcome was finding the cause of hypoglycaemia during the follow-up period.

### Statistical analysis

Data are presented as mean ± standard deviation (SD) for continuous variables and as frequencies (%) for categorical variables. Incidence density sampling was used to assign an index date to the non-exposure group by matching the exposed to a sample of the non-exposed who were at risk at the exposures’ index date. The multiple imputation analysis was conducted for variables with missing data; it included BMI, HbA1c, smoking, and alcohol consumption, and these variables were used in the final analysis with 25 imputations to produce imputed data.

We matched each exposed patient with up to two non-exposed patients. We included baseline variables (including age, sex, BMI, HbA1c, smoking, and alcohol consumption), diabetes duration, hypoglycaemia (prior to the index date) chronic comorbidities (12 months before the index date), co-prescribed medications (6 months before the index date), and antidiabetic medication as confounding variables (all variables are presented in Table 1), and those were used to calculate the propensity scores. The balance achieved by matching propensity scores was assessed using standardized differences; an absolute standardized difference between study groups <0.1 was considered negligible.

**Table 1:** Population characteristics.

Characteristics	Exposure *N* = 15,470 (100%)	Non-exposure *N* = 118,194 (100%)	*p* value
Age at index date Mean (SD)	80.42 years (7.5)	65.14 years (10.6)	<0.001
Gender (male %)	6,922 (44.74)	66,483 (56.25)	<0.001
Diabetes duration Mean (SD)	12.59 years (9.1)	6.37 years (4.2)	<0.001
Follow-up time, person-years Mean (SD)	3.091 (2.06)	6.51 (4.14)	<0.001
**Diabetes types (%)**			< 0.001
Type 1 diabetes mellitus	549 (3.55)	1,465 (1.24)	
Type 2 diabetes mellitus	14,921 (96.45)	116,729 (98.76)	
**Dementia types (%)**			
Vascular dementia	4,891 (31.62)	–	–
Alzheimer’s disease	3,846 (24.86)	–	–
Parkinson’s disease dementia	75 (0.48)	–	–
Lewy body disease	155 (1)	–	–
Frontotemporal dementia	25 (0.1)	–	–
Posterior cortical atrophy	2 (0.01)	–	–
Unspecified	6,476 (41.86)	–	–
**BMI (%)**			<0.001
<18.5	409 (2.64)	370 (0.31)	
18.5–24.9	4,716 (30.48)	12,838 (10.86)	
25–29.9	5,182 (33.49)	35,720 (30.22)	
30–39.9	3,458 (22.35)	46,788 (39.59)	
≥40	307 (1.98)	8,937 (7.56)	
Missing	1,398 (9.04)	13,541 (11.46)	
**HbA1c (%)**			<0.001
<4	213 (1.77)	1,031 (0.87)	
4–5.6	2007 (12.97)	14,533 (12.29)	
5.7–6.4	2,594 (16.77)	16,011 (13.55)	
6.5–13.9	9,822 (63.50)	42,174 (35.68)	
≥14	80 (0.52)	483 (0.41)	
Missing	754 (4.82)	43,962 (37.19)	
**Smoking (%)**			<0.001
Non-smoker	5,850 (37.82)	39,238 (33.19)	
Ex-smoker	7,592 (49.08)	49,355 (41.26)	
Smoker	1,445 (9.34)	19,279 (16.31)	
Missing	583 (3.77)	10,342 (8.75)	
**Alcohol consumption (%)**			<0.001
Non-drinker	2,736 (17.69)	13,401 (11.34)	
Ex-drinker	3,471 (22.44)	14,990 (12.68)	
Drinker	7,394 (47.79)	73,144 (61.88)	
Missing	1869 (12.08)	16,659 (14.09)	
**Chronic comorbidities (%)**			
Chronic obstructive pulmonary disease	1,555 (10.05)	13,143 (11.12)	<0.001
Cerebrovascular disease	4,258 (27.52)	11,816 (10)	<0.001
Arrhythmias	3,536 (22.86)	15,913 (13.46)	<0.001
Myocardial infarction	6,133 (39.64)	35,833 (30.32)	<0.001
Hypertension	11,253 (72.74)	80,548 (68.15)	<0.001
Chronic kidney disease	6,198 (40.06)	24,339 (20.59)	<0.001
Obesity	2,124 (13.73)	26,221 (22.2)	<0.001
Depression	5,827 (37.67)	37,388 (31.63)	<0.001
**Diabetes microvascular complications (%)**			
Neuropathy	3,062 (19.79)	16,007 (13.54)	<0.001
Nephropathy	138 (0.89)	361 (0.31)	<0.001
Retinopathy	6,145 (39.72)	36,111 (30.55)	<0.001
Diabetic foot	1,267 (8.19)	2,999 (2.54)	<0.001
**Antidiabetic medication (%)**			
Insulin	3,962 (25.61)	14,905 (12.61)	<0.001
Sulfonylurea	8,979 (58.16)	47,581 (40.26)	<0.001
Metformin	12,406 (80.36)	110,686 (93.65)	<0.001
GLP-1	189 (4.49)	4,025 (3.41)	<0.001
SGLT-2	107 (0.69)	5,483 (4.64)	<0.001
Meglitinides	193 (1.25)	795 (0.67)	<0.001
TZD	2,021 (13.09)	10,397 (8.80)	<0.001
DDP-4	1,903 (12.33)	22,902 (19.38)	<0.001
Acarbose	298 (1.93)	392 (0.33)	<0.001
**Medications (%)**			
Beta-blockers	7,397 (47.82)	55,320 (46.80)	0.02
ACEI-ARBS	11,233 (72.61)	84,803 (71.75)	0.03
Calcium channel blockers	7,432 (48.04)	56,023 (47.40)	0.13
Statins	2,335 (15.09)	23,345 (19.75)	<0.001
Anti-dementia	4,746 (30.68)	–	–
Antidepressants	3,807 (24.61)	24,113 (20.40)	<0.001
Aspirin	11,596 (74.96)	62,652 (53.01)	<0.001
**Hypoglycaemia**			
History of hypoglycaemia	1,131 (7.31)	1,367 (1.16)	<0.001

SD, standard deviation; GLP-1, glucagon-like peptide-1 receptor agonists; SGLT-2, sodium-glucose cotransporter-2 inhibitors; TZD, thiazolidinedione; DDP-4, dipeptidyl peptidase-4 inhibitors; ACEI-ARBs, angiotensin-converting enzyme inhibitors/angiotensin receptor blockers.

Cox proportional hazards regression models were used for the PS-matched patients and all patients without PS matching to examine associations of dementia and hypoglycaemic events risk for all models. All results were expressed as hazard ratios (HRs) with the statistical significance level set at 95% confidence intervals, *p* < 0.05. All analyses were conducted using statistical software (SAS, version 9.4).

### Sensitivity analysis

Two sensitivity analyses were conducted to assess the robustness of the main results. Firstly, Cox proportional hazards regression full model was adjusted for all covariates mentioned above. By using the multivariate analysis, we looked at the various independent variables that influence the dependent variable. Secondly, a sensitivity analysis was conducted to address the unmeasured confounding issue using the *E*-value of our HR results (21). The *E*-value method estimated the minimum strength of the association that would be required between an unmeasured confounder and both dementia diagnosis and risk of hypoglycaemia, conditional on the measured covariates to explain away an observed association (21).

## Results

### Population characteristics

**Figure 2:**
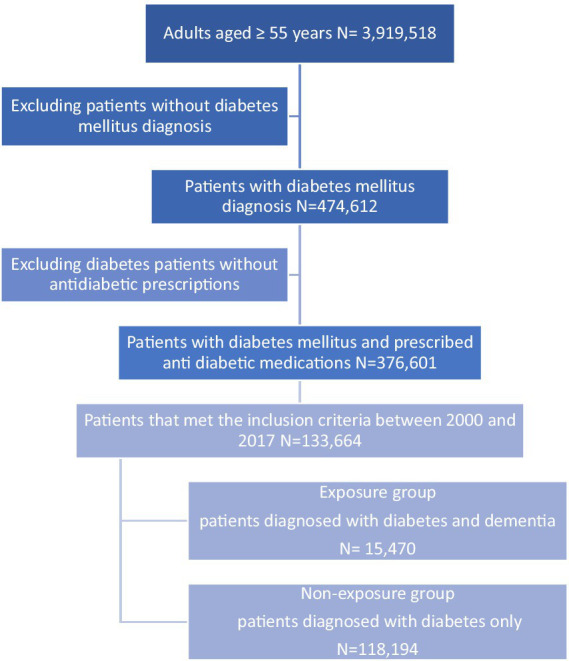
Flowchart of the study population.

In total, 133,664 diabetic patients were included in the cohort with a mean (SD) follow-up period of 6.11 (4.11) years. The mean (SD) age of our study population was 66.91 years (11.4) at the index date, and the mean (SD) of DM duration was 7.09 years (5.4). Men accounted for 73,405 (54.91%), and 131,650 patients (98.49%) were diagnosed with type 2 diabetes mellitus (T2DM) and 2,014 (1.51%) were patients with type 1 diabetes mellitus (T1DM). Among diabetic patients, 15,470 (11.57%) were diagnosed with dementia during the study period (Figure 2). A total of 24,442 hypoglycaemic episodes were experienced by 5,589 patients (4.18%) during the follow-up period. The baseline characteristics of the study population are presented in Table 1.

### Propensity score matching analysis

After propensity score matching, a total of 20,706 matched patients were identified, and 7,762 (37.48%) patients comprised the exposed group were matched with 12,944 (62.51%) non-exposed participants. The baseline characteristics of the matched group are shown in Table 2; all variables with absolute values of standardized differences were <0.1; accordingly, all confounding variables were considered properly adjusted by propensity score matching. The mean follow-up period of the matched pairs was 4.09 years. A total of 1,410 hypoglycaemic episodes were experienced during the study follow-up period (209 episodes per person per year experienced by the exposed group, compared to 164 episodes per person per year experienced by the non-exposed group). The adjusted hazard ratio (HR) for hypoglycaemic events for the exposed participants was (HR, 2.00; 95% CI, 1.63–2.66) compared with the non-exposed. In addition to dementia, we found other predictors of hypoglycaemia were age ≥65 years, being a man, T1DM, smoking, BMI, history of hypoglycaemic events, arrhythmia, and the use of insulin and sulfonylureas, which have a statistically significant effect on hypoglycaemia risk (*p*-value < 0.05; Table 3).

**Table 2:** Population characteristics after propensity score matching.

Characteristics	Exposure *N* = 7,762 (100%)	Non-exposure *N* = 12,944 (100%)	Standardized mean difference
Age at index date Mean (SD)	79.1 years (7.80)	78.7 years (8.40)	0.07
Gender (male %)	3,643 (46.82)	6,246 (48,25)	−0.03
Diabetes duration Mean (SD)	9.57 years (6.32)	9.24 years (4.66)	−0.02
Mean follow up time, person-years Mean (SD)	3.01 person-years (2.05)	4.75 person-years (3.35)	
**BMI**			0.07
<18.5	210 (2.70)	245 (1.89)	
18.5–24.9	2,350 (30.28)	3,687 (28.49)	
25–29.9	2,914 (37.55)	4,992 (38.56)	
30–39.9	2,076 (26.75)	3,637 (28.10)	
≥40	212 (2.73)	383 (2.96)	
**HbA1c**			0.01
<4	102 (1.32)	174 (1.34)	
4–5.6	1,072 (13.81)	1,837 (14.19)	
5.7–6.4	1,479 (19.05)	2,481 (19.17)	
6.5–13.9	5,058 (65.16)	8,368 (64.65)	
≥14	51 (0.66)	84 (0.65)	
**Diabetes types (%)**			−0.02
Type 1 diabetes mellitus	170 (2.19)	251 (1.94)	
Type 2 diabetes mellitus	7,592 (97.80)	12,693 (98.06)	
**Dementia types (%)**			
Vascular dementia	3,095 (39.88)	–	
Alzheimer’s disease	1,264 (16.28)	–	
Parkinson’s disease dementia	41 (0.52)	–	
Lewy body disease	50 (0.64)	–	
Frontotemporal dementia	18 (0.24)	–	
Posterior cortical atrophy	2 (0.03)	–	
Unspecified	3,292 (42.41)	–	
**Smoking (%)**			0.02
Non-smoker	2,932 (37.78)	4,780 (36.93)	
Ex-smoker	3,953(50.93)	6,670 (51.53)	
Smoker	877 (11.30)	1,494 (11.54)	
**Alcohol consumption (%)**			0.03
Non-drinker	1,528 (19.68)	2,392 (18.48)	
Ex-drinker	1,909 (24.59)	3,153 (24.35)	
Drinker	4,325 (55.73)	7,399 (57.16)	
**Chronic comorbidities (%)**			
Chronic obstructive pulmonary disease	908 (11.70)	1,566 (12.10)	−0.02
Cerebrovascular disease	2,084 (26.85)	3,307 (25.55)	0.03
Arrhythmias	1,884 (24.27)	3,140 (24.26)	0.003
Myocardial infarction	3,050 (39.29)	5,155 (39.83)	−0.01
Hypertension	5,646 (72.74)	9,477 (73.21)	−0.02
Chronic kidney disease	3,033 (39.07)	5,039 (38.93)	0.01
Obesity	1,120 (14.43)	1,910 (14.76)	−0.01
Depression	2,771 (35.70)	4,615 (35.65)	0.001
**Diabetes microvascular complications (%)**			
Neuropathy	1,472 (18.96)	2,409 (18.61)	0.01
Nephropathy	53 (0.68)	85 (0.66)	0.003
Retinopathy	2,844 (36.64)	4,706 (36.36)	0.01
Diabetic foot	560 (7.21)	828 (6.39)	−0.06
**Antidiabetic medication (%)**			
Insulin	1,565 (20.16)	2,524 (19.50)	0.03
Sulfonylurea	4,383 (56.47)	7,172 (55.41)	0.04
Metformin	6,403 (82.50)	10,653 (82.30)	0.04
GLP-1	176 (2.27)	295 (2.28)	0.03
SGLT-2	140 (1.80)	243 (1.88)	0.06
Meglitinides	80 (1.03)	168 (1.30)	0.04
TZD	1,030 (13.27)	1,716 (13.26)	0.01
DDP-4	1,443 (18.59)	2,416 (18.67)	−0.06
Acarbose	93 (1.20)	155 (1.19)	−0.02
**Medications (%)**			
Beta-blockers	3,879 (49.97)	6,669 (51.52)	−0.03
ACEI-ARBS	5,743 (73.99)	9,717 (75.07)	−0.02
Calcium channel blockers	3,834 (49.39)	6,512 (50.31)	−0.02
Statins	1,210 (15.59)	2,087 (16.12)	−0.01
Anti-dementia	298 (3.84)	–	–
Antidepressants	1,782 (22.96)	2,934 (22.67)	0.01
Aspirin	5,712 (73.50)	9,514 (73.50)	−0.001
**Hypoglycaemia**			
History of hypoglycaemia	329 (4.24)	509 (3.93)	0.02

SD, standard deviation; GLP-1, glucagon-like peptide-1 receptor agonists; SGLT-2, sodium-glucose cotransporter-2 inhibitors; TZD, thiazolidinedione; DDP-4, dipeptidyl peptidase-4 inhibitors; ACEI-ARBs, angiotensin-converting enzyme inhibitors/angiotensin receptor blockers.

**Table 3:** Cox proportional hazards ratio model and 95% confidence intervals for the risk of hypoglycaemia associated with dementia for PS-matched patients.

Variable	Propensity score matched
	Adjusted HR (95% CI)
**Age**	
<65 years	Reference
≥65 years	1.74 (1.03–3.04)
**Gender**	
Female	Reference
Male	1.07 (1.01–2.22)
**HbA1c (mmol/mol)**	
HbA1c = 4–5.7	Reference
HbA1c < 4	2.4 (2.1–3.4)
**Diabetes type**	
Type 2 diabetes mellitus	Reference
Type 1 diabetes mellitus	1.64 (1.01–2.64)
**Smoking**	
Non-smoker	Reference
Smoker	1.07 (1.07–2.11)
**BMI**	
18.5–24.9	Reference
<18.5	1.26 (1.06–1.61)
**Diabetes duration**	
Diabetes duration ≤5 years	Reference
Diabetes duration >5 years	2.4 (2.2–2.7)
**Hypoglycaemia**	
No history of hypoglycaemia	Reference
History of hypoglycaemia	3.2 (2.27–4.01)
**Antidiabetic medication**	
Insulin	2.07 (1.46–3.61)
Sulfonylurea	1.87 (1.03–2.98)

### Multivariable analysis

This analysis included 15,470 dementia and 118,194 non-dementia patients. Unadjusted results from the Cox proportional hazards regression models revealed a 2-fold (HR, 2.34; 95% CI, 2.32–2.39) increased risk for hypoglycaemia among diabetic patients diagnosed with dementia compared with those who did not develop dementia. Adjustment for baseline characteristics, chronic comorbidities, and medication use produced similar results (the multivariate-adjusted HR, 2.25; 95% CI, 2.22–2.32). Table 4 illustrates the results of the unadjusted and adjusted Cox proportional hazards regression models.

**Table 4:** Cox proportional hazards ratio models and 95% confidence intervals for the risk of hypoglycaemia associated with dementia (multivariable analysis).

Variable	Multivariable analysis	
	Unadjusted HR (95% CI)	Adjusted* HR (95% CI)
**Age**		
<65 years	Reference	Reference
≥65 years	1.90 (1.17–2.99)	1.87 (1.15–2.80)
**Gender**		
Female	Reference	Reference
Male	1.16 (1.10–2.04)	1.10 (1.06–2.01)
**HbA1c (mmol/mol)**		
HbA1c = 4–5.7	Reference	Reference
HbA1c < 4	3.1 (2.2–3.4)	2.6 (2.1–3.0)
**Diabetes type**		
Type 2 diabetes mellitus	Reference	Reference
Type 1 diabetes mellitus	1.68 (1.06–2.01)	1.60 (1.03–2.00)
**Smoking**		
Non-smoker	Reference	Reference
Smoker	1.10 (1.02–2.01)	1.07 (1.001–1.91)
**BMI**		
18.5–24.9	Reference	Reference
<18.5	1.30 (1.11–2.04)	1.28 (1.08–1.99)
**Diabetes duration**		
Diabetes duration ≤5 years	Reference	Reference
Diabetes duration >5 years	2.8 (2.2–3.1)	2.6 (2.1–2.9)
**Hypoglycaemia**		
No history of hypoglycaemia	Reference	Reference
History of hypoglycaemia	3.55 (3.2–5.64)	3.40 (3.00–5.59)
Insulin	2.14 (1.63–2.96)	2.09 (1.59–2.75)
Sulfonylurea	1.96 (1.29–3.01)	1.89 (1.19–2.97)

*For multivariable analysis, adjusted for baseline characteristics (age, gender, body mass index, smoking status, alcohol consumption, HbA1C, diabetes duration and history of hypoglycaemic events), chronic comorbidities (diabetes microvascular complications (including neuropathy, nephropathy, retinopathy and diabetic foot), hypertension, myocardial infarction, heart failure, chronic obstructive pulmonary disease, chronic kidney disease, depression, cerebrovascular disease, lipoedema and obesity), and medication use.

### Sensitivity analysis

The *E*-value for the point estimate and upper confidence bound for the risk of hypoglycaemia were 3.41 and 2.64, respectively.

## Discussion

This population-based retrospective cohort study showed that diabetic patients with a diagnosis of dementia had a double risk of experiencing hypoglycaemia compared with non-dementia diabetic patients. The association remained even after adjustment for age, sex, BMI, and other covariates.

Consistent with our results, previous trials and observational studies showed that dementia is an important risk factor for hypoglycaemia (13, 22–25). In the prospective population-based Health, Aging, and Body Composition Study, the risk of hypoglycaemia was assessed, and it was found that a 3-fold increased risk was significantly associated with severe cognitive dysfunction (HR 3.1; 95% CI, 1.5–6.6). However, they adjusted only for age, sex, education, insulin use, race/ethnicity prevalent DM, baseline score of the Modified Mini-Mental State Examination (3MS), and three comorbidities (MI, stroke, and hypertension), and they involved only 783 elderly patients who had DM diagnosis, which is a relatively small sample size (13). Furthermore, a nested case–control study in the UK using the CPRD database found that a 2-fold increase in the risk of severe hypoglycaemia was associated with dementia among newly treated T2DM (adjusted OR 2.10; 95% CI, 1.35–3.25) (26).

Varied results may possibly be due to differences in study design, population characteristics, frequency, or severity of hypoglycaemia.

However, to our knowledge, no known prior cohort study has retrospectively estimated the risk of hypoglycaemia among patients diagnosed with both DM and dementia using the IMRD-UK healthcare database.

Hypoglycaemia is the most frequent side effect associated with DM management, and the risk increases with age (27). Our study showed that the age (≥65 years) of patients diagnosed with both DM and dementia was significantly associated with an increased risk of hypoglycaemia (HR, 1.74; 95% CI, 1.03–3.04). Patients with cognitive impairment or dementia may experience a higher rate of hypoglycaemia due to several factors including unawareness or unrecognition of the warning symptoms of hypoglycaemic events, reduced secretion of glucagon, and altered psychomotor performance resulting in an inability to make decisions and control hypoglycaemia correctly (12, 28). In addition to age and dementia diagnosis, other predictors were found to be significantly associated with the risk of hypoglycaemia including being a man, having T1DM, very low BMI, smoking, and history of hypoglycaemia. A history of hypoglycaemia was associated with a 3-fold increased risk of experiencing hypoglycaemia in patients diagnosed with DM and dementia. A history of hypoglycaemia could induce a reversible pathophysiological state called hypoglycaemia unawareness state with hypoglycaemia-associated autonomic failure (an inefficient homeostatic glucose compensatory mechanism leading to neurogenic responses) (29).

All antidiabetic medications have a hypoglycaemic effect. However, certain classes with statistical significance tend to have a higher risk for hypoglycaemia, particularly SFU and insulin, which may be inappropriate to be prescribed for the elderly with dementia (30).

Our study findings highlight the importance of cognitive function and hypoglycaemia in the clinical management of DM among the elderly. The greater risk for hypoglycaemia associated with dementia among the diabetic population may reflect challenges and difficulties in DM self-management (13). Healthcare professionals should be aware and careful in managing DM among older patients diagnosed with dementia as they are at a higher risk of experiencing hypoglycaemia (30, 31). Additionally, family relatives/carers need to know the warning symptoms and management of hypoglycaemia by undergoing an educational program that may help to support and improve the patient’s quality of life.

### Strength and limitations

This study has several strengths, including a population-based observational study design with a long follow-up period and a large cohort. Hypoglycaemia definition was based on diagnostic codes (Read codes), which is a more objective measure than self-report. We have applied the multiple imputation techniques to address the missing data, which was considered to be a good approach when analysing large datasets with missing data in the literature (32, 33). We were also able to minimize bias by using the propensity score matching technique to balance the risk differences at baseline. However, several study limitations also should be considered. First, this was a retrospective analysis, and the database had incomplete blood results which may have affected the results, particularly patients with milder or moderate hypoglycaemia. Although our dementia definition for the exposed group was likely very specific, it was probably insensitive to mild cases of cognitive impairment. Moreover, most diagnostic codes of dementia entered were non-specific and did not allow us to differentiate between subtypes. Second, due to the observational design nature of the study, unmeasured confounding variables may have persisted despite PS or multivariable analyses. Importantly, the sensitivity analysis using the *E*-value methodology suggested that the observed HR of 2.00 for hypoglycaemia could only be explained by an unmeasured confounder that was associated with both dementia and risk of hypoglycaemia by a risk ratio of more than 3.41 above and beyond that of the confounders that were measured in this study (upper confidence bound, 2.64). The unmeasured confounders risk ratio such as age, diabetes duration, chronic comorbidities, antidiabetic medications, and history of hypoglycaemia is much greater than any observed for known hypoglycaemia risk factors examined in the present study. It is unlikely that the unmeasured or unknown confounder by having a relative risk exceeding 3.41, would have a greater significant effect on hypoglycaemia than the variables observed well-known risk factors of hypoglycaemia.

## Conclusion

In summary, our results provide evidence for a significant association between dementia and hypoglycaemia among the diabetes population in the UK from 2000 to 2017. Reduced cognitive function in patients with dementia may increase the risk of hypoglycaemic events. Dementia and hypoglycaemia should be considered in the clinical management of the elderly diagnosed with DM. Further studies are needed to identify the risk factors and effective management strategies to reduce the frequency of hypoglycaemia among the elderly diagnosed with DM and dementia, as well as in those with milder forms of cognitive impairment.

## Data availability statement

The data analyzed in this study is subject to the following licenses/restrictions: data available only upon reasonable request. Requests to access these datasets should be directed to AA, aaalsharif@pnu.edu.sa.

## Ethics statement

The studies involving human participants were reviewed and approved by this study was reviewed and scientific approval was obtained by IQVIA Scientific Review Committee in 2018 (18THIN046). Written informed consent for participation was not required for this study in accordance with the national legislation and the institutional requirements.

## Author contributions

AA and LW: conceptualization, data curation, investigation, project administration, resources, validation, and writing original draft. AA, TM, HA, and LW: formal analysis. AA, HA, and LW: methodology. LW: supervision. AA, IW, TM, WL, MA, HA, and LW: writing—review and editing. All authors contributed to the article and approved the submitted version.

## Conflict of interest

The authors declare that the research was conducted in the absence of any commercial or financial relationships that could be construed as a potential conflict of interest.

## Publisher’s note

All claims expressed in this article are solely those of the authors and do not necessarily represent those of their affiliated organizations, or those of the publisher, the editors and the reviewers. Any product that may be evaluated in this article, or claim that may be made by its manufacturer, is not guaranteed or endorsed by the publisher.
